# Crystal structure of the co-crystalline adduct 1,3,6,8-tetra­aza­tri­cyclo­[4.4.1.1^3,8^]dodecane (TATD)–4-chloro-3,5-di­methyl­phenol (1/1)

**DOI:** 10.1107/S2056989015010257

**Published:** 2015-06-03

**Authors:** Augusto Rivera, Jicli José Rojas, Jaime Ríos-Motta, Michael Bolte

**Affiliations:** a Universidad Nacional de Colombia, Sede Bogotá, Facultad de Ciencias, Departamento de Química, Cra 30 No. 45-03, Bogotá, Código Postal 111321, Colombia; b Institut für Anorganische Chemie, J. W. Goethe-Universität Frankfurt, Max-von Laue-Strasse 7, 60438 Frankfurt/Main, Germany

**Keywords:** crystal structure, co-crystalline adducts, TATD, hydrogen bonding, C—H⋯π inter­actions

## Abstract

In the crystal of the title co-crystalline adduct, C_8_H_16_N_4_·C_8_H_9_ClO, (I), prepared by solid-state reaction, the mol­ecules are linked by inter­molecular O—H⋯N hydrogen bonds, forming a *D* motif. The aza­adamantane structure in (I) is slightly distorted, with N—CH_2_—CH_2_—N torsion angles of 10.4 (3) and −9.0 (3)°. These values differ slightly from the corresponding torsion angles in the free aminal cage (0.0°) and in related co-crystalline adducts, which are not far from a planar geometry and consistent with a *D*_2*d*_ mol­ecular symmetry in the tetra­aza­tri­cyclo structure. The structures also differ in that there is a slight elongation of the N—C bond lengths about the N atom that accepts the hydrogen bond in (I) compared with the other N—C bond lengths. In the crystal, the two mol­ecules are not only linked by a classical O—H⋯N hydrogen bond but are further connected by weak C—H⋯π inter­actions, forming a two-dimensional supra­molecular network parallel to the *bc* plane.

## Abstract 2

In the crystal of the title co-crystalline adduct, the 1,3,6,8-tetra­aza­tri­cyclo­[4.4.1.1^3,8^]dodecane (TATD) and 4-chloro-3,5-di­methyl­phenol are linked by inter­molecular O—H⋯N hydrogen bonds and C—H⋯π inter­actions.

## Chemical context   

In our continuing investigations on the reactivity of cyclic aminals of the adamantane type with phenols, we have found that 1,3,6,8-tetra­aza­tri­cyclo­[4.4.1.1^3,8^]dodecane (TATD) shows an inter­esting reactivity with 4-chloro-3,5-di­methyl­phenol under different conditions. Reaction between TATD with 4-chloro-3,5-di­methyl­phenol in solution yields symmetrical bis-benzoxazines (Rivera *et al.*, 2005 ▸), but under heating in an oil bath (Rivera & Quevedo, 2013 ▸) or microwave-assisted solvent-free conditions, symmetrical *N,N*’-disubstituted imidazolidines (Rivera, Nerio & Bolte, 2015 ▸) are obtained. Therefore, we became inter­ested in exploring the reactivity of TATD with phenols under solvent-free conditions at room temperature. In the course of our investigations, we obtained the mol­ecular salt 8,10,12-tri­aza-1-azonia­tetra­cyclo[8.3.1.1^8,12^.0^2,7^]penta­decane 4-nitro­phenolate 4-nitro­phenol by grinding (2*R*,7*R*)-1,8,10,12-tetra­aza­tetra­cyclo­[8.3.1.1^8,12^.0^2,7^]penta­decane with 4-nitro­phenol (Rivera, Uribe, Ríos-Motta *et al.*, 2015 ▸) and the 1:2 adduct 1,3,6,8-tetra­aza­tri­cyclo[4.4.1.1^3,8^]dodecane (TATD)-4-bromo­phenol (Rivera, Uribe, Rojas *et al.*, 2015 ▸) by grinding at room temperature.
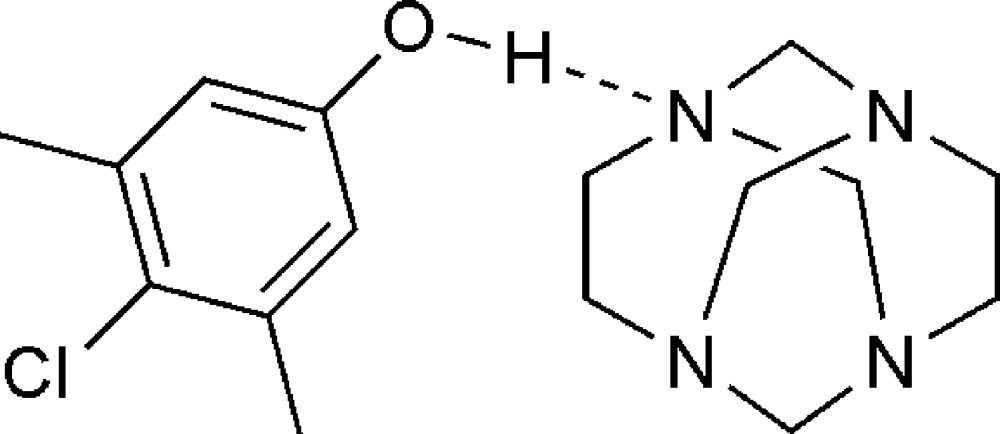


 Herein, we describe the synthesis of the title co-crystalline adduct 1,3,6,8-tetra­aza­tri­cyclo­[4.4.1.1^3,8^]dodecane (TATD)–4-chloro-3,5-di­methyl­phenol under solvent-free conditions by simply grinding together the components at room temperature.

## Structural commentary   

**Figure 1:**
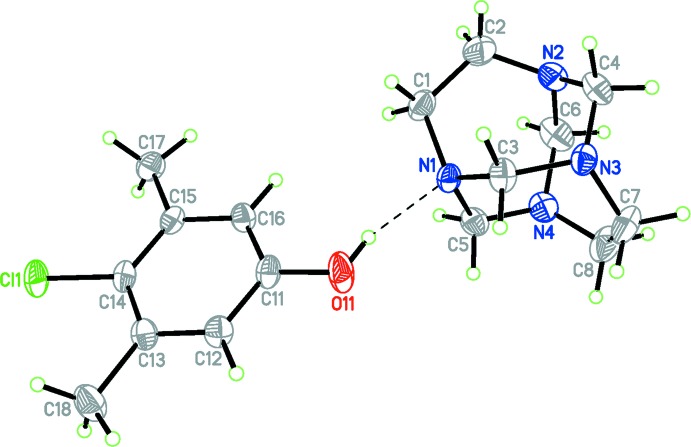
Perspective view of the title compound, with displacement ellipsoids drawn at the 50% probability level. The hydrogen bond is drawn as a dashed line.

The crystal structure of the title compound, (I), has confirmed the presence of a 1:1 co-crystalline adduct. A view of this adduct is shown in Fig. 1 ▸. The asymmetric unit of the title compound contains a 1,3,6,8-tetra­aza­tri­cyclo­[4.4.1.1^3,8^]dodecane (TATD) and a 4-chloro-3,5-di­methyl­phenol mol­ecule linked *via* an O—H⋯N hydrogen bond, forming a *D* motif (Bernstein *et al.*, 1995 ▸). As in the 1:2 adduct with 4-bromo­phenol (Rivera, Uribe, Rojas *et al.*, 2015 ▸) and the 1:1 adduct with hydro­quinone (Rivera *et al.*, 2007 ▸), the inter­molecular O—H⋯N hydrogen bond in (I) also leads to a stable supra­molecular structure, but comparison of the title compound with the above-mentioned related structures shows that the three adducts differ in the O⋯N hydrogen-bond distances [2.752 (2) Å in (I), 2.705 (5) Å in the 1:2 adduct and 2.767 (2) Å in the co-crystalline adduct with hydro­quinone], which is in agreement with the differences in the p*K*a values between the species involved in the hydrogen bond (Majerz *et al.*, 1997 ▸): 4-chloro-3,5-di­methyl­phenol (p*K*a = 9.76); *p*-bromo­phenol (p*K*a = 9.37) and hydro­quinone (p*K*a = 9.85) (Lide, 2003 ▸).

To a first approximation, the geometric parameters of the title mol­ecule agree well with those reported for similar structures (Rivera *et al.*, 2007 ▸; Rivera, Uribe, Rojas *et al.*, 2015 ▸) and are within normal ranges (Allen *et al.*, 1987 ▸), but compared to the free aminal cage structure (Rivera *et al.*, 2014 ▸) which belongs to the *D_2d_* point group, two small differences are noted. The aza­adamantane structure in (I) is slightly distorted, with N—CH_2—_CH_2_—N torsion angles of 10.4 (3)° (N1—C1—C2—N2) and −9.0 (3)° (N3—C7—C8—N4). These values differ slightly from the values of the corresponding torsion angles in the free aminal cage (0.0°; Rivera *et al.*, 2014 ▸), and the related co-crystalline adducts [2.4 (7)° (Rivera, Uribe, Rojas *et al.*, 2015 ▸) and −0.62° (Rivera *et al.*, 2007 ▸)] which shows that each N—C—C—N group is not far from a planar geometry and consistent with a *D_2d_* mol­ecular symmetry in the tetra­aza­tri­cyclo structure. Furthermore, the structures also differ in the slight elongation of the N1—C bond lengths of the nitro­gen atom that accepts the hydrogen bond, [1.470 (2) and 1.480 (2) Å], compared to the the other N—C bond lengths (Table 1 ▸).

## Supra­molecular features   

**Figure 2:**
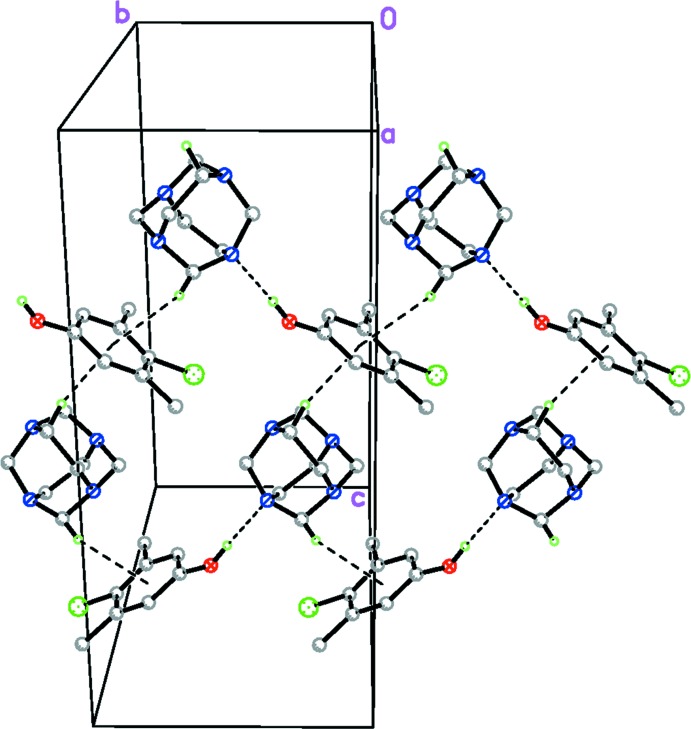
Packing diagram of the title compound. Only H atoms involved in hydrogen bonding are shown. Hydrogen bonds are drawn as dashed lines.

**Figure 3:**
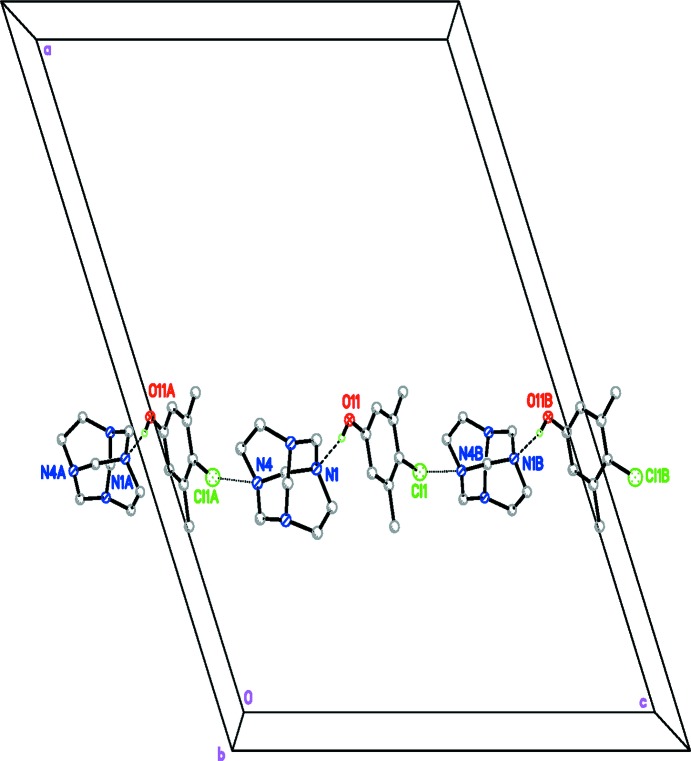
Partial packing diagram of the title compound, viewed along the *b* axis. Only H atoms involved in hydrogen bonding are shown. Hydrogen bonds are drawn as dashed lines and the short Cl⋯N contacts are shown as dotted lines. Atoms with suffix A are generated by the symmetry operator (*x*, −*y*, *z* − 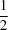) and atoms with suffix B are generated by the symmetry operator (*x*, −*y*, *z* + 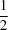).

The two different mol­ecules in (I) are connected by a classical O—H⋯N hydrogen bond. The crystal packing is further stabilized by weak inter­molecular C—H⋯π inter­actions, linking the mol­ecules into two-dimensional sheets in the *bc* plane (Table 2 ▸ and Fig. 2 ▸). Furthermore, there are short N⋯Cl contacts [N4⋯Cl1^i^ 3.1680 (15) Å; symmetry operator: (i) *x*, −*y*, *z* − 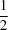] linking the mol­ecules into zigzag chains running along the *c-*axis direction (Fig. 3 ▸).

## Database survey   

The geometric parameters of 4-chloro-3,5-di­methyl­phenol in (I) (Table 1 ▸) agree well with those of found in the crystal structure containing only this mol­ecule (Cox, 1995 ▸), which crystallized with two mol­ecules in the asymmetric unit [C—O = 1.387 (3) and 1.378 (3) Å; C—Cl = 1.752 (2) and 1.749 (2) Å; C—C_meth­yl_ = 1.502 (3), 1.500 (3), 1.514 (3) and 1.505 (3) Å]. For 1,3,6,8-tetra­aza­tri­cyclo­[4.4.1.1^3,8^]dodecane, two comparable structures were retrieved from the CSD (Groom & Allen, 2014 ▸). A least-squares fit of the structure that contains only 1,3,6,8-tetra­aza­tri­cyclo­[4.4.1.1^3,8^]dodecane (Rivera *et al.*, 2014 ▸) gives an r.m.s. deviation of 0.048 Å with 1,3,6,8-tetra­aza­tri­cyclo­[4.4.1.1^3,8^]dodecane of (I) and a least-squares fit of 1,3,6,8-tetra­aza­tri­cyclo­[4.4.1.1^3,8^]dodecane co-crystallized with hydro­quinone (Rivera *et al.*, 2007 ▸) gives an r.m.s. deviation of 0.051 Å with 1,3,6,8-tetra­aza­tri­cyclo­[4.4.1.1^3,8^]dodecane of (I). Thus, it can be concluded that the conformational freedom of 1,3,6,8-tetra­aza­tri­cyclo­[4.4.1.1^3,8^]dodecane is rather limited.

## Synthesis and crystallization   

A mixture of 1,3,6,8-tetra­aza­tri­cyclo­[4.4.1.^3,8^]dodecane (TATD) (168 mg, 1 mmol) and 4-chloro-3,5-di­methyl­phenol (157 mg, 1 mmol) was ground using a mortar and pestle, at room temperature for 15 min., as required to complete the reaction (TLC). The mixture was then dissolved in methanol. Crystals suitable for X-ray diffraction were obtained from a methanol solution upon slow evaporation of the solvent at room temperature.

## Refinement   

Crystal data, data collection and structure refinement details are summarized in Table 3 ▸. All H atoms were located in difference electron-density maps. The hydroxyl H atom was refined freely, while C-bound H atoms were fixed geometrically (C—H = 0.95, 0.98 or 0.99 Å) and refined using a riding model, with *U*_iso_(H) values set at 1.2*U*_eq_ (1.5 for methyl groups) of the parent atom. The methyl groups were allowed to rotate but not to tip.

## Supplementary Material

Crystal structure: contains datablock(s) I. DOI: 10.1107/S2056989015010257/zs2335sup1.cif

Structure factors: contains datablock(s) I. DOI: 10.1107/S2056989015010257/zs2335Isup2.hkl

Supporting information file. DOI: 10.1107/S2056989015010257/zs2335Isup3.cml

CCDC reference: 1403518

Additional supporting information:  crystallographic information; 3D view; checkCIF report

## Figures and Tables

**Table 1:** Selected geometric parameters (, )

N1C1	1.470(2)	N3C7	1.455(2)
N1C5	1.470(2)	N3C4	1.458(2)
N1C3	1.480(2)	N4C5	1.444(2)
N2C2	1.449(3)	N4C6	1.456(2)
N2C6	1.454(3)	N4C8	1.457(3)
N2C4	1.462(2)	Cl1C14	1.7534(16)
N3C3	1.446(2)	O11C11	1.356(2)
			
N1C1C2N2	10.4(3)	N3C7C8N4	9.0(3)

**Table 2:** Hydrogen-bond geometry (, ) *Cg*8 is the centroid of the C11C16 ring.

*D*H*A*	*D*H	H*A*	*D* *A*	*D*H*A*
O11H11N1	0.85(4)	1.92(4)	2.752(2)	165(3)
C3H3*A* *Cg*8^i^	0.99	2.89	3.837(2)	160
C8H8*A* *Cg*8^ii^	0.99	2.88	3.814(2)	157

Symmetry codes: (i) 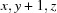; (ii) 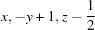.

**Table 3:** Experimental details

Crystal data	
Chemical formula	C_8_H_16_N_4_C_8_H_9_ClO
*M*_r_	324.85
Crystal system, space group	Monoclinic, *C*2/*c*
Temperature (K)	173
*a*, *b*, *c* ()	25.6048(18), 7.5295(7), 18.2317(13)
()	111.080(5)
*V* (^3^)	3279.7(5)
*Z*	8
Radiation type	Mo *K*
(mm^1^)	0.24
Crystal size (mm)	0.27 0.26 0.22

Data collection	
Diffractometer	Stoe *IPDS* II two-circle
Absorption correction	Multi-scan (*X-RED32*; Stoe Cie, 2001 ▸)
*T*_min_, *T*_max_	0.738, 1.000
No. of measured, independent and observed [*I* > 2(*I*)] reflections	14414, 3066, 2512
*R*_int_	0.083
(sin /)_max_ (^1^)	0.608

Refinement	
*R*[*F* ^2^ > 2(*F* ^2^)], *wR*(*F* ^2^), *S*	0.040, 0.107, 1.02
No. of reflections	3066
No. of parameters	205
H-atom treatment	H atoms treated by a mixture of independent and constrained refinement
_max_, _min_ (e ^3^)	0.26, 0.25

Computer programs: *X-AREA* (Stoe Cie, 2001 ▸), *SHELXS97* and *XP* in *SHELXTL-Plus* (Sheldrick, 2008 ▸) and *SHELXL2014* (Sheldrick, 2015 ▸).

## supplementary crystallographic information

### Crystal data

**Table:**

C_8_H_16_N_4_·C_8_H_9_ClO	*F*(000) = 1392
*M**_r_* = 324.85	*D*_x_ = 1.316 Mg m^−^^3^
Monoclinic, *C*2/*c*	Mo *K*α radiation, λ = 0.71073 Å
*a* = 25.6048 (18) Å	Cell parameters from 12525 reflections
*b* = 7.5295 (7) Å	θ = 3.4–25.8°
*c* = 18.2317 (13) Å	µ = 0.24 mm^−^^1^
β = 111.080 (5)°	*T* = 173 K
*V* = 3279.7 (5) Å^3^	Block, colourless
*Z* = 8	0.27 × 0.26 × 0.22 mm

### Data collection

**Table:**

Stoe IPDS II two-circle diffractometer	2512 reflections with *I* > 2σ(*I*)
Radiation source: Genix 3D IµS microfocus X-ray source	*R*_int_ = 0.083
ω scans	θ_max_ = 25.6°, θ_min_ = 3.4°
Absorption correction: multi-scan (*X-RED32*; Stoe & Cie, 2001)	*h* = −30→30
*T*_min_ = 0.738, *T*_max_ = 1.000	*k* = −9→9
14414 measured reflections	*l* = −19→22
3066 independent reflections	

### Refinement

**Table:**

Refinement on *F*^2^	0 restraints
Least-squares matrix: full	Hydrogen site location: mixed
*R*[*F*^2^ > 2σ(*F*^2^)] = 0.040	H atoms treated by a mixture of independent and constrained refinement
*wR*(*F*^2^) = 0.107	*w* = 1/[σ^2^(*F*_o_^2^) + (0.0635*P*)^2^ + 0.5777*P*] where *P* = (*F*_o_^2^ + 2*F*_c_^2^)/3
*S* = 1.02	(Δ/σ)_max_ < 0.001
3066 reflections	Δρ_max_ = 0.26 e Å^−^^3^
205 parameters	Δρ_min_ = −0.25 e Å^−^^3^

### Special details

**Table:**

Geometry. All e.s.d.'s (except the e.s.d. in the dihedral angle between two l.s. planes) are estimated using the full covariance matrix. The cell e.s.d.'s are taken into account individually in the estimation of e.s.d.'s in distances, angles and torsion angles; correlations between e.s.d.'s in cell parameters are only used when they are defined by crystal symmetry. An approximate (isotropic) treatment of cell e.s.d.'s is used for estimating e.s.d.'s involving l.s. planes.

### Fractional atomic coordinates and isotropic or equivalent isotropic displacement parameters (Å^2^)

**Table:**

	*x*	*y*	*z*	*U*_iso_*/*U*_eq_	
N1	0.37029 (5)	0.56339 (18)	0.36779 (8)	0.0224 (3)	
N2	0.30414 (6)	0.8359 (2)	0.26828 (9)	0.0297 (3)	
N3	0.40890 (6)	0.84503 (19)	0.33226 (9)	0.0260 (3)	
N4	0.35050 (6)	0.58696 (19)	0.22295 (9)	0.0299 (3)	
C1	0.31697 (7)	0.6087 (2)	0.37648 (11)	0.0313 (4)	
H1A	0.3246	0.6345	0.4326	0.038*	
H1B	0.2924	0.5030	0.3623	0.038*	
C2	0.28501 (8)	0.7651 (3)	0.32812 (14)	0.0438 (5)	
H2A	0.2455	0.7287	0.3022	0.053*	
H2B	0.2857	0.8627	0.3649	0.053*	
C3	0.41163 (7)	0.7098 (2)	0.38985 (10)	0.0267 (4)	
H3A	0.4084	0.7700	0.4363	0.032*	
H3B	0.4494	0.6559	0.4068	0.032*	
C4	0.35575 (8)	0.9394 (2)	0.29998 (12)	0.0333 (4)	
H4A	0.3578	1.0180	0.2575	0.040*	
H4B	0.3524	1.0170	0.3419	0.040*	
C5	0.36300 (7)	0.4777 (2)	0.29227 (11)	0.0285 (4)	
H5A	0.3977	0.4111	0.2989	0.034*	
H5B	0.3325	0.3894	0.2818	0.034*	
C6	0.30356 (8)	0.7083 (3)	0.20810 (12)	0.0376 (5)	
H6A	0.2997	0.7761	0.1599	0.045*	
H6B	0.2693	0.6355	0.1961	0.045*	
C7	0.43347 (9)	0.7933 (3)	0.27484 (12)	0.0386 (5)	
H7A	0.4705	0.7391	0.3032	0.046*	
H7B	0.4399	0.9020	0.2487	0.046*	
C8	0.39926 (10)	0.6640 (3)	0.21162 (13)	0.0430 (5)	
H8A	0.3865	0.7265	0.1604	0.052*	
H8B	0.4241	0.5661	0.2084	0.052*	
Cl1	0.35337 (2)	−0.26689 (6)	0.61168 (3)	0.03412 (16)	
O11	0.43692 (5)	0.32897 (19)	0.47753 (9)	0.0390 (4)	
H11	0.4116 (15)	0.396 (5)	0.447 (2)	0.084 (10)*	
C11	0.41519 (7)	0.1923 (2)	0.50578 (10)	0.0257 (4)	
C12	0.45255 (7)	0.0659 (2)	0.55103 (10)	0.0261 (4)	
H12	0.4913	0.0774	0.5597	0.031*	
C13	0.43430 (7)	−0.0768 (2)	0.58371 (10)	0.0250 (4)	
C14	0.37708 (7)	−0.0899 (2)	0.56943 (10)	0.0237 (3)	
C15	0.33830 (7)	0.0318 (2)	0.52312 (10)	0.0229 (3)	
C16	0.35825 (7)	0.1742 (2)	0.49162 (10)	0.0245 (4)	
H16	0.3327	0.2597	0.4601	0.029*	
C17	0.27635 (7)	0.0110 (2)	0.50600 (12)	0.0311 (4)	
H17A	0.2638	−0.1047	0.4813	0.047*	
H17B	0.2559	0.1057	0.4704	0.047*	
H17C	0.2692	0.0185	0.5552	0.047*	
C18	0.47576 (8)	−0.2148 (3)	0.63056 (13)	0.0388 (5)	
H18A	0.4719	−0.2311	0.6817	0.058*	
H18B	0.5139	−0.1753	0.6384	0.058*	
H18C	0.4684	−0.3277	0.6019	0.058*	

### Atomic displacement parameters (Å^2^)

**Table:**

	*U*^11^	*U*^22^	*U*^33^	*U*^12^	*U*^13^	*U*^23^
N1	0.0202 (6)	0.0238 (7)	0.0228 (7)	−0.0040 (5)	0.0074 (5)	0.0018 (5)
N2	0.0289 (7)	0.0269 (7)	0.0311 (8)	0.0034 (6)	0.0080 (6)	0.0026 (6)
N3	0.0285 (7)	0.0250 (7)	0.0264 (8)	−0.0061 (6)	0.0122 (6)	−0.0009 (6)
N4	0.0394 (8)	0.0270 (7)	0.0227 (8)	−0.0012 (6)	0.0104 (6)	−0.0047 (6)
C1	0.0261 (8)	0.0386 (10)	0.0328 (10)	−0.0012 (7)	0.0152 (8)	0.0041 (8)
C2	0.0350 (10)	0.0483 (11)	0.0558 (14)	0.0129 (9)	0.0259 (10)	0.0134 (10)
C3	0.0243 (8)	0.0289 (8)	0.0233 (9)	−0.0075 (6)	0.0042 (7)	0.0006 (7)
C4	0.0402 (10)	0.0206 (8)	0.0367 (11)	0.0003 (7)	0.0108 (8)	0.0009 (7)
C5	0.0331 (9)	0.0206 (8)	0.0309 (10)	−0.0020 (6)	0.0106 (7)	−0.0034 (7)
C6	0.0410 (10)	0.0353 (10)	0.0247 (10)	0.0032 (8)	−0.0024 (8)	−0.0014 (8)
C7	0.0408 (10)	0.0466 (11)	0.0376 (11)	−0.0112 (8)	0.0252 (9)	−0.0016 (9)
C8	0.0609 (13)	0.0429 (11)	0.0379 (12)	−0.0081 (10)	0.0333 (11)	−0.0079 (9)
Cl1	0.0372 (2)	0.0318 (2)	0.0342 (3)	−0.00899 (18)	0.01382 (19)	0.00941 (18)
O11	0.0250 (6)	0.0406 (8)	0.0481 (9)	−0.0075 (6)	0.0090 (6)	0.0216 (7)
C11	0.0256 (8)	0.0271 (8)	0.0240 (9)	−0.0081 (7)	0.0084 (7)	0.0023 (7)
C12	0.0194 (7)	0.0308 (9)	0.0256 (9)	−0.0054 (6)	0.0049 (6)	0.0012 (7)
C13	0.0267 (8)	0.0260 (8)	0.0197 (8)	−0.0027 (6)	0.0051 (7)	0.0003 (6)
C14	0.0298 (8)	0.0231 (8)	0.0189 (8)	−0.0077 (6)	0.0096 (7)	−0.0009 (6)
C15	0.0251 (8)	0.0253 (8)	0.0201 (8)	−0.0051 (6)	0.0102 (6)	−0.0028 (6)
C16	0.0240 (8)	0.0256 (8)	0.0220 (8)	−0.0011 (6)	0.0062 (7)	0.0033 (7)
C17	0.0255 (9)	0.0332 (9)	0.0373 (11)	−0.0038 (7)	0.0145 (7)	0.0008 (8)
C18	0.0325 (9)	0.0385 (10)	0.0387 (11)	0.0028 (8)	0.0047 (8)	0.0122 (9)

### Geometric parameters (Å, º)

**Table:**

N1—C1	1.470 (2)	C7—C8	1.522 (3)
N1—C5	1.470 (2)	C7—H7A	0.9900
N1—C3	1.480 (2)	C7—H7B	0.9900
N2—C2	1.449 (3)	C8—H8A	0.9900
N2—C6	1.454 (3)	C8—H8B	0.9900
N2—C4	1.462 (2)	Cl1—C14	1.7534 (16)
N3—C3	1.446 (2)	O11—C11	1.356 (2)
N3—C7	1.455 (2)	O11—H11	0.85 (4)
N3—C4	1.458 (2)	C11—C12	1.391 (2)
N4—C5	1.444 (2)	C11—C16	1.393 (2)
N4—C6	1.456 (2)	C12—C13	1.388 (2)
N4—C8	1.457 (3)	C12—H12	0.9500
C1—C2	1.521 (3)	C13—C14	1.396 (2)
C1—H1A	0.9900	C13—C18	1.512 (2)
C1—H1B	0.9900	C14—C15	1.391 (2)
C2—H2A	0.9900	C15—C16	1.397 (2)
C2—H2B	0.9900	C15—C17	1.511 (2)
C3—H3A	0.9900	C16—H16	0.9500
C3—H3B	0.9900	C17—H17A	0.9800
C4—H4A	0.9900	C17—H17B	0.9800
C4—H4B	0.9900	C17—H17C	0.9800
C5—H5A	0.9900	C18—H18A	0.9800
C5—H5B	0.9900	C18—H18B	0.9800
C6—H6A	0.9900	C18—H18C	0.9800
C6—H6B	0.9900		
			
C1—N1—C5	113.12 (13)	N4—C6—H6B	107.5
C1—N1—C3	113.50 (13)	H6A—C6—H6B	107.0
C5—N1—C3	114.75 (13)	N3—C7—C8	115.84 (15)
C2—N2—C6	114.25 (16)	N3—C7—H7A	108.3
C2—N2—C4	113.62 (17)	C8—C7—H7A	108.3
C6—N2—C4	114.37 (16)	N3—C7—H7B	108.3
C3—N3—C7	114.43 (15)	C8—C7—H7B	108.3
C3—N3—C4	115.49 (14)	H7A—C7—H7B	107.4
C7—N3—C4	114.98 (16)	N4—C8—C7	115.87 (15)
C5—N4—C6	115.37 (15)	N4—C8—H8A	108.3
C5—N4—C8	114.79 (15)	C7—C8—H8A	108.3
C6—N4—C8	114.58 (16)	N4—C8—H8B	108.3
N1—C1—C2	116.37 (15)	C7—C8—H8B	108.3
N1—C1—H1A	108.2	H8A—C8—H8B	107.4
C2—C1—H1A	108.2	C11—O11—H11	112 (2)
N1—C1—H1B	108.2	O11—C11—C12	117.13 (15)
C2—C1—H1B	108.2	O11—C11—C16	123.30 (16)
H1A—C1—H1B	107.3	C12—C11—C16	119.58 (15)
N2—C2—C1	117.66 (15)	C13—C12—C11	121.22 (15)
N2—C2—H2A	107.9	C13—C12—H12	119.4
C1—C2—H2A	107.9	C11—C12—H12	119.4
N2—C2—H2B	107.9	C12—C13—C14	117.86 (15)
C1—C2—H2B	107.9	C12—C13—C18	119.89 (15)
H2A—C2—H2B	107.2	C14—C13—C18	122.22 (15)
N3—C3—N1	118.95 (14)	C15—C14—C13	122.59 (15)
N3—C3—H3A	107.6	C15—C14—Cl1	118.87 (12)
N1—C3—H3A	107.6	C13—C14—Cl1	118.54 (13)
N3—C3—H3B	107.6	C14—C15—C16	117.92 (14)
N1—C3—H3B	107.6	C14—C15—C17	121.63 (15)
H3A—C3—H3B	107.0	C16—C15—C17	120.45 (15)
N3—C4—N2	118.66 (14)	C11—C16—C15	120.81 (15)
N3—C4—H4A	107.6	C11—C16—H16	119.6
N2—C4—H4A	107.6	C15—C16—H16	119.6
N3—C4—H4B	107.6	C15—C17—H17A	109.5
N2—C4—H4B	107.6	C15—C17—H17B	109.5
H4A—C4—H4B	107.1	H17A—C17—H17B	109.5
N4—C5—N1	118.91 (13)	C15—C17—H17C	109.5
N4—C5—H5A	107.6	H17A—C17—H17C	109.5
N1—C5—H5A	107.6	H17B—C17—H17C	109.5
N4—C5—H5B	107.6	C13—C18—H18A	109.5
N1—C5—H5B	107.6	C13—C18—H18B	109.5
H5A—C5—H5B	107.0	H18A—C18—H18B	109.5
N2—C6—N4	119.39 (15)	C13—C18—H18C	109.5
N2—C6—H6A	107.5	H18A—C18—H18C	109.5
N4—C6—H6A	107.5	H18B—C18—H18C	109.5
N2—C6—H6B	107.5		
			
C5—N1—C1—C2	−73.2 (2)	C3—N3—C7—C8	74.7 (2)
C3—N1—C1—C2	59.8 (2)	C4—N3—C7—C8	−62.5 (2)
C6—N2—C2—C1	59.7 (2)	C5—N4—C8—C7	−63.3 (2)
C4—N2—C2—C1	−74.0 (2)	C6—N4—C8—C7	73.6 (2)
N1—C1—C2—N2	10.4 (3)	N3—C7—C8—N4	−9.0 (3)
C7—N3—C3—N1	−79.67 (19)	O11—C11—C12—C13	178.74 (17)
C4—N3—C3—N1	57.2 (2)	C16—C11—C12—C13	−1.2 (3)
C1—N1—C3—N3	−84.10 (19)	C11—C12—C13—C14	0.3 (3)
C5—N1—C3—N3	48.1 (2)	C11—C12—C13—C18	178.17 (17)
C3—N3—C4—N2	−51.5 (2)	C12—C13—C14—C15	1.1 (3)
C7—N3—C4—N2	85.2 (2)	C18—C13—C14—C15	−176.72 (17)
C2—N2—C4—N3	78.8 (2)	C12—C13—C14—Cl1	−178.85 (13)
C6—N2—C4—N3	−54.8 (2)	C18—C13—C14—Cl1	3.3 (2)
C6—N4—C5—N1	−51.7 (2)	C13—C14—C15—C16	−1.5 (2)
C8—N4—C5—N1	84.85 (19)	Cl1—C14—C15—C16	178.46 (13)
C1—N1—C5—N4	79.28 (19)	C13—C14—C15—C17	177.67 (16)
C3—N1—C5—N4	−53.1 (2)	Cl1—C14—C15—C17	−2.4 (2)
C2—N2—C6—N4	−83.3 (2)	O11—C11—C16—C15	−179.15 (17)
C4—N2—C6—N4	50.0 (2)	C12—C11—C16—C15	0.8 (3)
C5—N4—C6—N2	56.6 (2)	C14—C15—C16—C11	0.5 (3)
C8—N4—C6—N2	−80.1 (2)	C17—C15—C16—C11	−178.67 (17)

### Hydrogen-bond geometry (Å, º)

Cg8 is the centroid of the C11–C16 ring.

**Table:**

*D*—H···*A*	*D*—H	H···*A*	*D*···*A*	*D*—H···*A*
O11—H11···N1	0.85 (4)	1.92 (4)	2.752 (2)	165 (3)
C3—H3*A*···*Cg*8^i^	0.99	2.89	3.837 (2)	160
C8—H8*A*···*Cg*8^ii^	0.99	2.88	3.814 (2)	157

Symmetry codes: (i) *x*, *y*+1, *z*; (ii) *x*, −*y*+1, *z*−1/2.

## References

[bb1] Allen, F. H., Kennard, O., Watson, D. G., Brammer, L., Orpen, A. G. & Taylor, R. (1987). *J. Chem. Soc. Perkin Trans. 2*, pp. S1–19.

[bb2] Bernstein, J., Davis, R. E., Shimoni, L. & Chang, N.-L. (1995). *Angew. Chem. Int. Ed. Engl.* **34**, 1555–1573.

[bb3] Cox, P. J. (1995). *Acta Cryst.* C**51**, 1361–1364.

[bb4] Groom, C. R. & Allen, F. H. (2014). *Angew. Chem. Int. Ed.* **53**, 662–671. 10.1002/anie.201306438 24382699

[bb5] Lide, D. R. (2003). *CRC Handbook of Chemistry and Physics*. Boca Raton, Florida: CRC Press.

[bb6] Majerz, I., Malarski, Z. & Sobczyk, L. (1997). *Chem. Phys. Lett.* **274**, 361–364.

[bb7] Rivera, A., Nerio, L. S. & Bolte, M. (2015). *Acta Cryst.* E**71**, 312–314. 10.1107/S2056989015002212 PMC4350744 25844196

[bb8] Rivera, A. & Quevedo, R. (2013). *Tetrahedron Lett.* **54**, 1416–1420.

[bb9] Rivera, A., Ríos-Motta, J. & Bolte, M. (2014). *Acta Cryst.* E**70**, o266. 10.1107/S1600536814002608 PMC3998434 24764981

[bb10] Rivera, A., Ríos-Motta, J., Hernández-Barragán, A. & Joseph-Nathan, P. (2007). *J. Mol. Struct.* **831**, 180–186.

[bb11] Rivera, A., Ríos-Motta, J., Quevedo, R. & Joseph-Nathan, P. (2005). *Rev. Colomb. Quim* **34**, 105-115.

[bb12] Rivera, A., Uribe, J. M., Ríos-Motta, J., Osorio, H. J. & Bolte, M. (2015). *Acta Cryst.* C**71**, 284–288. 10.1107/S2053229615004829 25836286

[bb13] Rivera, A., Uribe, J. M., Rojas, J. J., Ríos-Motta, J. & Bolte, M. (2015). *Acta Cryst.* E**71**, 463–465. 10.1107/S2056989015006684 PMC4420134 25995856

[bb14] Sheldrick, G. M. (2008). *Acta Cryst.* A**64**, 112–122. 10.1107/S0108767307043930 18156677

[bb15] Sheldrick, G. M. (2015). *Acta Cryst.* C**71**, 3–8.

[bb16] Stoe & Cie (2001). *X-AREA* and *X-RED32*. Stoe & Cie, Darmstadt, Germany.

